# Supplementary material for: A Simple and Effective Phosphine-Doping Technique for Solution-Processed Nanocrystal Solar Cells
Source: Nanomaterials (Basel). 2023 May 30;13(11):1766. doi: 10.3390/nano13111766 (PMC10254249; doi:10.3390/nano13111766)

Supplementary Materials

Chenbo Min [1], Yihui Chen [1], Yonglin Yang [1], Hongzhao Wu [1], Bailin Guo [1], Sirui Wu [1], Qichuan Huang [1] and Donghuan Qin [1,2,*] and Lintao Hou [3]

Table S1. Summarized XPS values of different elements for two types of films

CdTe	C 1s	Cd 3d	O 1s	Te 3d	P 2p
**BE (eV)**	281.90	401.96	527.20	572.80	129.50
**Atomic concentration (%)**	36.8	17.7	32.6	12.4	0.4

CdTe with TPP (5000ppm)	C 1s	Cd 3d	O 1s	Te 3d	P 2p
**BE (eV)**	281.70	401.96	527.20	572.90	135.50
**Atomic concentration (%)**	43.3	17.5	25.5	10.2	3.6

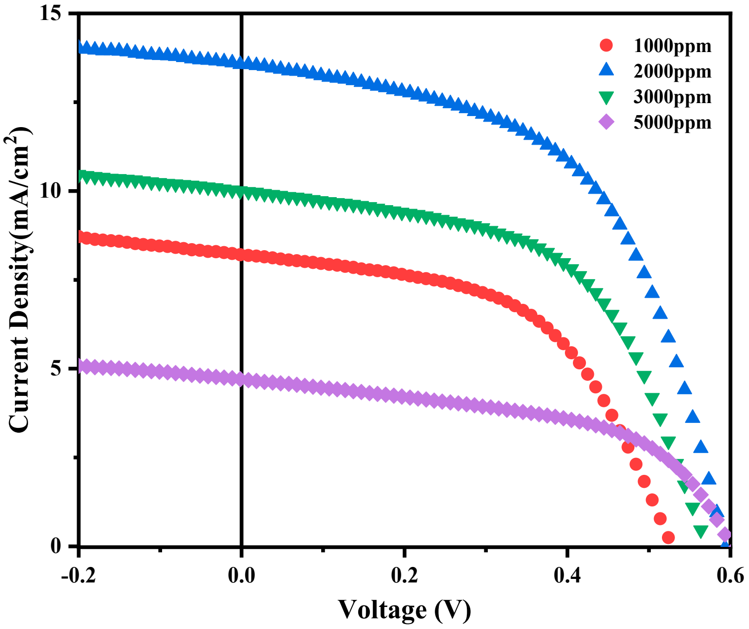

Figure S1. *J–V* curves of ITO/ZnO/CdSe/CdTe/TPP/Au with different doping concentration.

Table S2. Summarized performance of NC solar cells with different doping concentration. (Figure S1).

Doping (ppm)	V_{oc} (V)	J_{sc} (mA/cm^2)	FF (%)	PCE (%)	R_s (Ω·cm^2)	R_{sh} (Ω·cm^2)
**1000**	0.52	8.21	53.78	2.31	19.12	351.14
**2000**	0.59	13.55	54.43	4.38	9.91	377.52
**3000**	0.57	10.00	55.11	3.16	15.24	435.86
**5000**	0.60	4.70	52.41	1.49	21.27	382.16

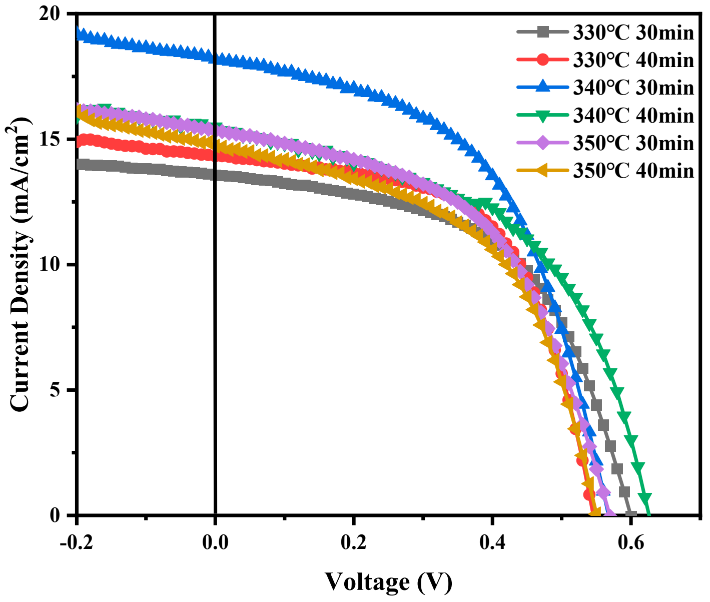

Figure S2. *J–V* curves of ITO/ZnO/CdSe/CdTe/TPP/Au with different temperature and annealing time.

Table S3. Summarized performance of NC solar cells with different heated time and temperature (Figure S2).

Device	V_{oc} (V)	J_{sc} (mA/cm²)	FF (%)	PCE (%)	R_s (Ω·cm²)	R_{sh} (Ω·cm²)
**330°C / 30min**	0.59	13.55	54.43	4.38	9.91	377.52
**330°C / 40min**	0.54	14.32	59.53	4.61	7.36	353.02
**340°C / 30min**	0.56	18.19	53.16	5.41	8.14	182.87
**340°C / 40min**	0.62	15.49	51.73	4.97	8.30	208.86
**350°C / 30min**	0.56	15.33	52.59	4.52	10.59	205.06
**350°C / 40min**	0.55	14.72	52.51	4.23	8.58	156.24

Figure S3. transient photovoltage (TPV) measurements of CdTe NC solar cells with different doping concentration.

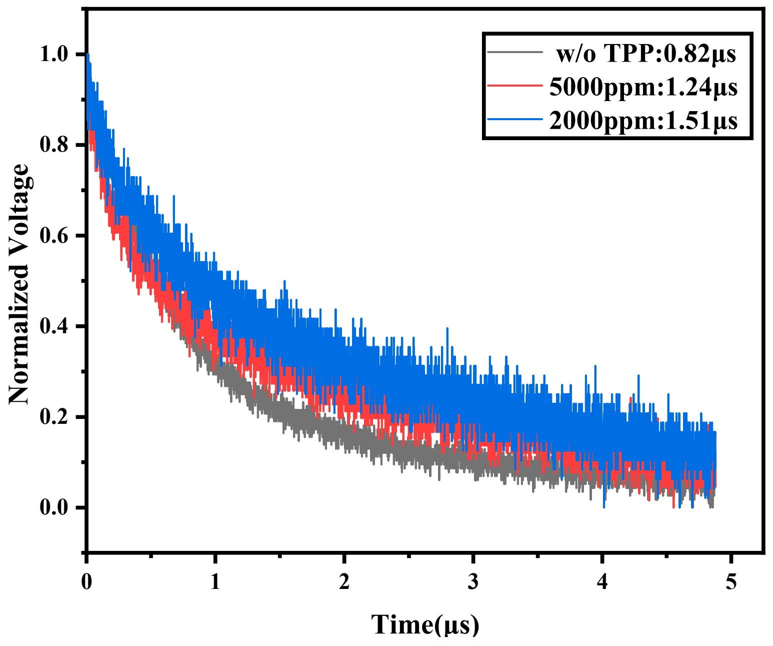

Supplement: Supplementary file 1 [file nanomaterials-13-01766-s001.zip › nanomaterials-2364988-supplementary.pdf]
